# RBM8A Depletion Decreases the Cisplatin Resistance and Represses the Proliferation and Metastasis of Breast Cancer Cells via AKT/mTOR Pathway

**DOI:** 10.1155/2022/4576789

**Published:** 2022-08-28

**Authors:** Tao Song, Huazhou Zhang

**Affiliations:** Department of General Surgery, The Second Affiliated Hospital of Xi'an Medical University, Xi'an City, Shaanxi Province 710000, China

## Abstract

**Background:**

Breast cancer (BC) is the most prevalent malignancy in women. This study is aimed to explore the role and regulatory mechanism of RNA-binding motif protein 8A (RBM8A) in BC.

**Methods:**

We detected the expression of RBM8A in BC tissues and cell lines (MCF-7, MDA-MB-231, and MDA-MB-436), and explored the correlation of RBM8A expression with clinicopathological features in patients. The function of RBM8A deficiency in MCF-7 and MDA-MB-231 cells was determined using MTT, wound healing, and transwell assay. The effect of RBM8A suppression on the cisplatin (DDP) resistance in MCF-7 and MDA-MB-231 cells was also evaluated. Besides, western blotting was used to examine AKT/mTOR pathway-related proteins. The mouse model was constructed to confirm the effect of RBM8A on tumor growth.

**Results:**

The expression of RBM8A was elevated in BC tissues and cell lines. RBM8A silencing restrained the malignant behaviors of MCF-7 and MDA-MB-231 cells, including viability, migration, and invasion, while promoting apoptosis. Silencing of RBM8A overcame resistance to DDP in MCF-7 and MDA-MB-231 cells. Furthermore, RBM8A suppression restrained the activation of the AKT/mTOR pathway in both MCF-7 and MDA-MB-231 cells. Feedback experiments revealed that SC79 treatment reversed the reduction effects of RBM8A knockdown on viability, DDP resistance, migration, and invasion of MDA-MB-231 cells. Moreover, the silencing of RBM8A inhibited the growth of tumor xenograft *in vivo*.

**Conclusions:**

RBM8A knockdown may reduce DDP resistance in BC to repress the development of BC via the AKT/mTOR pathway, suggesting that RBM8A may serve as a new therapeutic target in BC.

## 1. Introduction

Breast cancer (BC) is one of the most frequently diagnosed malignancies among women across the globe [1]. The incidence and mortality of BC are increasing each year [2]. The incidence of BC rises with age until menopause, and BC is more aggressive in younger females [3]. Despite the therapeutic strategies for BC have been improved greatly, patients still suffer from some adverse effects such as poor outcomes and drug-resistance [4]. Therefore, it is essential to explore the pathogenesis of BC and find a new and effective therapeutic target for BC treatment.

RNA-binding motif protein 8A (RBM8A) is an RNA-binding motif protein widely expressed in cells and involved in the regulation of cell proliferation, metastasis, apoptosis, and other biological functions [5, 6]. In addition, it is involved in various crucial cell signaling pathways and plays an important role in tumorigenesis and development [7, 8]. Importantly, abnormal expression of RBM8A is closely associated with a variety of malignancies. For instance, Liang et al. have reported that RBM8A overexpression can enhance the resistance to oxaliplatin in hepatocellular cancer [9]. RBM8A suppression retards the proliferation of mesothelioma cells [10]. In addition, gastric cancer patients with increased RBM8A expression have lower overall survival [11]. However, the function and underlying mechanism of RBM8A in BC is still unknown.

Abundant evidence has indicated that the AKT/mTOR pathway plays a pivotal role in different gynecological cancers via modulating cellular processes and drug resistance. Overexpression of Derlin1 has been shown to promote cell proliferation and migration and induce apoptosis by activating the AKT/mTOR pathway in cervical cancer [12]. The activation of the AKT/mTOR pathway has been reported to reduce cisplatin (DDP)-induced apoptosis, thereby leading to cisplatin resistance in ovarian cancer [13]. Notably, the AKT/mTOR pathway is also involved in the progression of BC. Wei et al. have reported that Magnoflorine retards BC growth and elevates the sensitivity of BC cells to doxorubicin (DOX) treatment by suppressing the AKT/mTOR pathway [14]. Inhibition of the AKT/mTOR pathway restrains the adipocyte-mediated proliferation and migration of BC cells [15]. In addition, Ren et al. have indicated that the activation of the AKT/mTOR pathway is involved in the proliferation, migration, and invasion of BC cells [16]. However, whether RBM8A interacts with the AKT/mTOR pathway in BC remains undefined.

In the current study, the expression of RBM8A was determined in BC and cell lines. The effects of RBM8A on the malignant behaviors and DDP resistance of BC cells were evaluated and further analyzed its expression level with clinicopathological features and prognosis. We then explored whether RBM8A regulates the AKT/mTOR pathway to affect BC progression. This research may uncover the molecular mechanism of RBM8A in BC and provide a new target for BC treatment.

## 2. Material and Methods

### 2.1. Clinical Samples

Seventy BC patients who underwent mastectomy were recruited from May 2016 to April 2019 at our hospital. BC tissues (*n* = 70) and the corresponding normal tissues (*n* = 70) were stored at −80°C. Prior to mastectomy, no radiotherapy or chemotherapy treatment was administered to the patients. All procedures involving human participants were in accordance with the ethical standards of the institutional and/or national research committee and with the 1964 Helsinki declaration and its later amendments or comparable ethical standards. The Medical Ethics Committee of the Second Affiliated Hospital of Xi'an Medical University approved this study and written informed consent was acquired from each participant.

### 2.2. Cell Culture

Human breast epithelial cell line MCF10A and three BC cell lines MCF-7, MDA-MB-436 (triple-negative), and MDA-MB-231 (triple-negative) were obtained from American Type Culture Collection (Manassas, VA, USA) and cultured in RPMI-1640 plus 10% FBS (Invitrogen, Carlsbad, CA, USA), 100 IU/mL penicillin, and 100 *μ*g/mL streptomycin (Sigma-Aldrich, St. Louis, MO, USA) at 37°C with 5% CO_2_.

### 2.3. Cell Transfection

The short hairpin (sh)-RBM8A (sh1-RBM8A and sh2-RBM8A) and sh-negative control (NC) were synthesized by GenePharma (Shanghai, China). MCF-7 and MDA-MB-231 cells grown to 80% confluence were transfected with these above agents using Lipofectamine 3000 reagent (Invitrogen). Approximately 48 h later, MCF-7 and MDA-MB-231 cells were used for further studies.

### 2.4. Quantitative Real-Time Polymerase Chain Reaction (qRT-PCR)

Total RNA was extracted with TRIzol reagent (Invitrogen). cDNA was generated from RNA template using a reverse transcription kit (Invitrogen). qRT-PCR analysis was performed by SYBR® Premix Ex Tap Reagent kit (TaKaRa, Dalian, China) [17]. *β*-actin was used for the normalization of RBM8A. The primer sequences are shown in Table 1. The relative expressions were calculated by using 2^−ΔΔCt^ method.

### 2.5. Western Blotting

Cells were lysed with lysis buffer, and proteins were extracted using a protein extraction kit (Beyotime, Shanghai, China) according to the manufacturer's instructions. The proteins were separated by electrophoresis in 10% sodium dodecyl sulfate-polyacrylamide gel electrophoresis and were electrotransferred onto polyvinylidene difluoride membranes. The membranes were blocked for 2 h with 5% non-fat milk at room temperature. Then, the membranes were incubated with primary antibodies against RBM8A (1 : 1000, HPA018403, Sigma-Aldrich), mTOR (1 : 1000, SAB4501039, Sigma-Aldrich), p-mTOR (1 : 1000, SAB4301415, Sigma-Aldrich), p-AKT (1 : 1000, SAB4504331, Sigma-Aldrich), AKT (1 : 1000, SAB4500797, Sigma-Aldrich), and *β*-actin (1 : 5000, SAB2701711, Sigma-Aldrich) at 4°C overnight. The membranes were then incubated with HRP conjugated secondary antibody (1 : 5000, 12–348, Sigma-Aldrich) for 1 h at room temperature. The immunoblots were measured by chemiluminescence detection system and quantified by ImageLab software (Bio-Rad, Hercules, CA, USA).

### 2.6. Cell Viability Assay

The MCF-7 and MDA-MB-231 cells were seeded into 96-well plates (2 × 10^3^ cells/well) and cultured with 5% CO_2_ at 37°C. MTT (0.5 mg/ml; Sigma-Aldrich) was added on 1, 2, 3, 4, and 5 days, respectively, and incubated for 4 h at 37°C. The absorbance at 490 nm was detected using a microplate reader (Bio-Rad). Additionally, the effects of DDP on BC cells were also evaluated by cell viability. Briefly, MCF-7 and MDA-MB-231 cells at logarithmic growth phase were seeded into 96-well plates (1 × 10^5^ cells/well). DDP was added at different concentrations (0, 2, 4, 6, 8, 10, 12, 14, 16, and 18 *μ*mol/L), followed by incubation for 48 h. Then, cell viability was measured as above described.

### 2.7. EdU Proliferation Assay

The proliferation of MCF-7 and MDA-MB-231 cells was assessed using an EdU assay kit (Ribobio, Guangzhou, China) based on the manufacturer's instructions. Briefly, the cells were first cultured with 50 *μ*M·EdU for 2 h at 37°C, followed by fixing with 4% formaldehyde, permeabilizing using 0.5% Triton X-100 for 20 min, and incubating with 1 × Apollo reaction cocktail for 30 min at room temperature. After that, DAPI (4′, 6-diamidino2-phenylindole) was utilized to stain DNA for another 30 min. The EdU-positive cells were observed under a fluorescence microscope (Carl Zeiss, Oberkochen, Germany).

### 2.8. Flow Cytometry Analysis

The apoptosis of MCF-7 and MDA-MB-231 cells was evaluated using the Annexin V-FITC apoptosis detection kit (Thermo Fisher Scientific) in accordance with the manufacturer's protocol. Briefly, 2 × 10^5^ cells were re-suspended in 500 *μ*l binding buffer and stained with Annexin V-FIPC-A and PE-A (both 5 *μ*l) at 4°C for 15 min in the dark. Subsequently, cell apoptosis was assessed using a FACScan flow cytometer (Becton, Dickinson and Company, Franklin Lakes, NJ, USA).

### 2.9. Wound Healing Assay

MCF-7 and MDA-MB-231 cells (1 × 10^6^/well) were incubated in 6-well plates. The cell monolayer was then wounded with 10-*μ*l pipette tip and cultured in serum-free medium. The width of the scratch was measured at 0 and 24 h.

### 2.10. Transwell Assay

Invasion assay was performed using the 24-well invasion chamber system pre-coated with 50 *μ*l Matrigel (Sigma-Aldrich). MCF-7 and MDA-MB-231 cells (1 × 10^5^ cells/well) suspended in serum-free medium were seeded into the upper chamber. The low chamber was filled with culture medium supplemented with 10% FBS. The invaded cells were dyed using crystal violet after 24 h incubation, and then, were counted under an inverted microscope (Olympus, Tokyo, Japan). For the measurement of migration, MCF-7 and MDA-MB-231 cells were seeded into the non-coated upper chamber, and the other steps were same as the above described.

### 2.11. Tumor Xenografts in Nude Mice

Female BALB/c nude mice (6 weeks) were purchased from Esebio (Shanghai, China). All procedures were in accordance with the ethical standards of the institution and approved by the Animal Care and Use Committee of the Second Affiliated Hospital of Xi'an Medical University. MDA-MB-231 cells (5 × 10^6^ cells) infected with lentivirus containing sh1-RBM8A or sh-NC were subcutaneously injected into the right axillary region of the mice (*n* = 6). The tumor volume was measured every five days and calculated with the formula: ^1^/_2_ LW^2^ (L, length; W, width). After 30 days post-cell injection, mice were anesthetized and then sacrificed by cervical dislocation, and tumor weight was tested. In addition, the tumor xenograft was collected for determination of RBM8A protein expression.

### 2.12. Statistical Analysis

Data were presented as mean ± standard deviation (SD) and analyzed by GraphPad Prism 8.0 statistical software (La Jolla, CA, USA). The differences between two groups (normally distributed) were assessed using Student's *t*-test. The differences among multiple groups were analyzed by one-way ANOVA followed by Tukey's multiple comparison test. Differences were considered statistically significant at *P* < 0.05.

## 3. Results

### 3.1. RBM8A Expression Is Increased in BC Cells and Tissues

**Figure 1:**
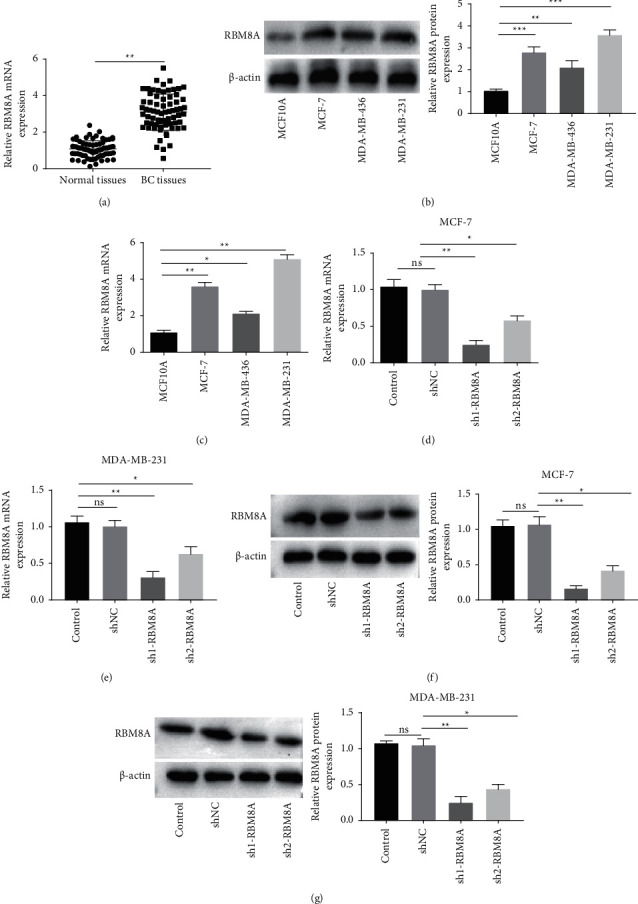
RBM8A expression is increased in breast cancer (BC) cells and tissues. (a) QRT-PCR was performed to detect the expression of RBM8A in BC tissues and paired normal tissues. ^*∗∗*^*P* < 0.01 vs. normal tissues; (b) western blot was performed to measure the protein expression of RBM8A in MCF10A, MCF-7, MDA-MB-436, and MDA-MB-231 cells. ^*∗∗*^*P* < 0.01, ^*∗∗∗*^*P* < 0.001 vs. MCF10A; (c) the expression of RBM8A in MCF10A, MCF-7, MDA-MB-436, and MDA-MB-231 cells was detected by qRT-PCR. ^*∗*^*P* < 0.05, ^*∗∗*^*P* < 0.01 vs. MCF10A; (d and e) the expression of RBM8A in MCF-7 and MDA-MB-231 cells was assessed by qRT-PCR; (f and g) the protein level of RBM8A in MCF-7 and MDA-MB-231 cells was determined by western blotting. ^*∗*^*P* < 0.05, ^*∗∗*^*P* < 0.01 vs. sh-NC. Each experiment was repeated three times.

As shown in Figure 1(a), RBM8A expression in BC tissues was higher than that in paired normal tissues (*P* < 0.01). We further assessed the correlation between RBM8A expression and clinicopathological features of 70 BC patients (Table 2). Our results illustrated that the high and low expression of RBM8A showed significant differences in TNM stage (*P* < 0.01) and lymph node metastasis (LNM) (*P* < 0.05). Compared with MCF10A cells, RBM8A protein expression was extremely enhanced in MCF-7, MDA-MB-436, and MDA-MB-231 cells (*P* < 0.01, Figure 1(b)). Additionally, RBM8A expression in MCF-7, MDA-MB-436, and MDA-MB-231 cells was higher than that in MCF10A cells (*P* < 0.05, Figure 1(c)). Afterward, the transfection efficiency of sh-RBM8A was determined. As shown in Figures 1(d)–1(e), RBM8A expression was remarkably decreased in BC cell lines transfected with sh1-RBM8A (*P* < 0.01) or sh2-RBM8A (*P* < 0.05). Similar patterns were observed in the results of RBM8A protein level measured by western blotting (*P* < 0.05, Figures 1(f)–1(g)). Therefore, sh1-RBM8A was chosen for the subsequent experiments due to the relatively high transfection efficiency.

### 3.2. RBM8A Knockdown Reduces Viability and DDP Resistance of BC Cells

**Figure 2:**
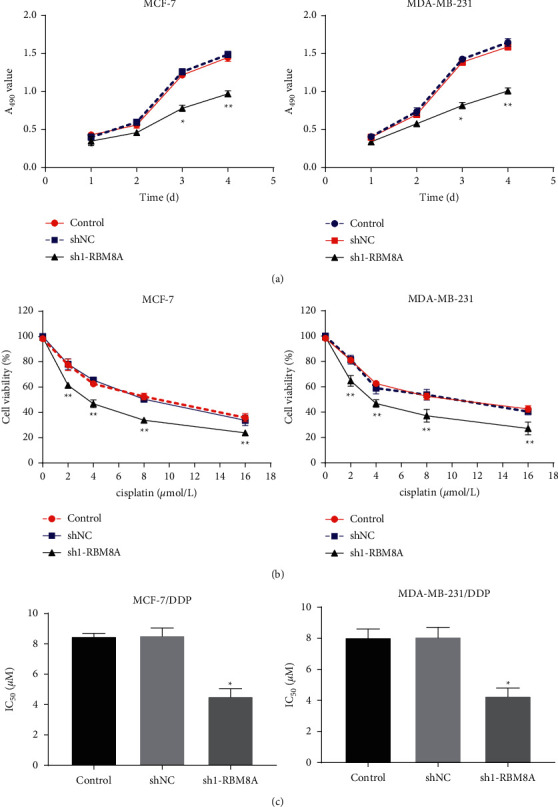
RBM8A knockdown reduces viability and cisplatin (DDP) resistance of breast cancer (BC) cells. (a) The viability of MCF-7 and MDA-MB-231 cells was analyzed by MTT  assay; (b) the viability of MCF-7 and MDA-MB-231 cells under different concentrations of DDP was evaluated by MTT assay. (c) IC50 of DDP was assessed. ^*∗*^*P* < 0.05, ^*∗∗*^*P* < 0.01 vs. sh-NC. Each experiment was repeated three times.

The effect of RBM8A knockdown on cell viability was then explored. As illustrated in Figure 2(a), we found that the viability of BC cells transfected with sh1-RBM8A was significantly decreased compared to those transfected with sh-NC (*P* < 0.05). Meanwhile, under the different concentrations of DDP treatment, the cell viability was also inhibited in the sh1-RBM8A group compared with the sh-NC group, suggesting that transfection of sh1-RBM8A enhanced the sensibility of BC cells to DDP (*P* < 0.01, Figure 2(b)). Additionally, DDP-resistant BC cells were transfected with sh1-RBM8A or sh-NC to further investigate the function of RBM8A in DDP-resistant BC. As demonstrated in Figure 2(c), RBM8A knockdown dramatically reduced the half-maximal inhibitory concentration (IC50) of DDP (*P* < 0.05).

### 3.3. Silencing of RBM8A Inhibits the Proliferation and Promotes the Apoptosis of BC Cells

**Figure 3:**
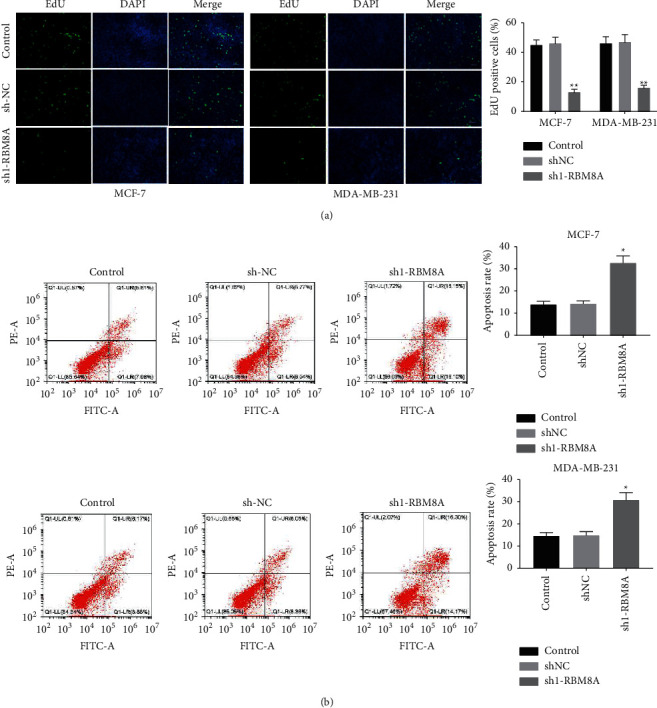
Silencing of RBM8A inhibits the proliferation and promotes the apoptosis of breast cancer (BC) cells. (a) The proliferation of MCF-7 and MDA-MB-231 cells was determined by EdU assay. (b) The apoptosis of MCF-7 and MDA-MB-231 cells was analyzed by flow cytometry assay. ^*∗*^*P* < 0.05, ^*∗∗*^*P* < 0.01 vs. sh-NC. Each experiment was repeated three times.

The direct impact of RBM8A on the proliferation and apoptosis of MCF-7 and MDA-MB-231 cells was also investigated. As shown in Figure 3(a), the EdU-positive cells in the sh1-RBM8A group were significantly reduced relative to the sh-NC group (*P* < 0.01), which confirmed that RBM8A silencing inhibited the proliferation of BC cells. Furthermore, we revealed that the apoptosis rate of BC cells transfected with sh1-RBM8A was remarkably elevated compared to those transfected with sh-NC (*P* < 0.05, Figure 3(b)).

### 3.4. RBM8A Knockdown Suppresses Migration and Invasion of BC Cells

**Figure 4:**
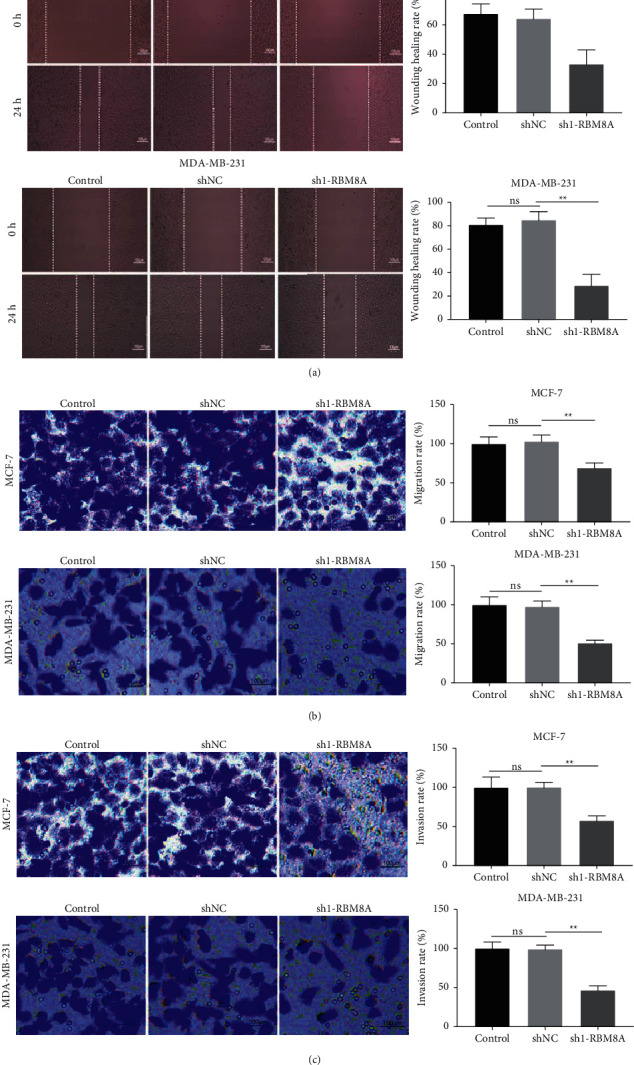
RBM8A knockdown suppresses migration and invasion of breast cancer (BC) cells. (a) Wound-healing assay was used to measure the migration of MCF-7 and MDA-MB-231 cells. ^*∗∗*^*P* < 0.01 vs. sh-NC; (b and c) the migration and invasion of MCF-7 and MDA-MB-231 cells were measured by transwell assay. ^*∗∗*^*P* < 0.01 vs. sh-NC. Each experiment was repeated three times.

The metastasis of BC cells was measured to further assess the role of RBM8A in BC. Wound-healing assay confirmed that RBM8A silencing markedly suppressed the migration of MCF-7 and MDA-MB-231 cells (*P* < 0.01, Figure 4(a)). Transwell assay also confirmed the inhibitory effect of RBM8A silencing for BC cell migration (*P* < 0.01, Figure 4(b)). Additionally, transwell assay also revealed that RBM8A knockdown could significantly repress the invasion of MCF-7 and MDA-MB-231 cells (*P* < 0.01, Figure 4(c)).

### 3.5. RBM8A Silencing Inhibits the AKT/mTOR Pathway

**Figure 5:**
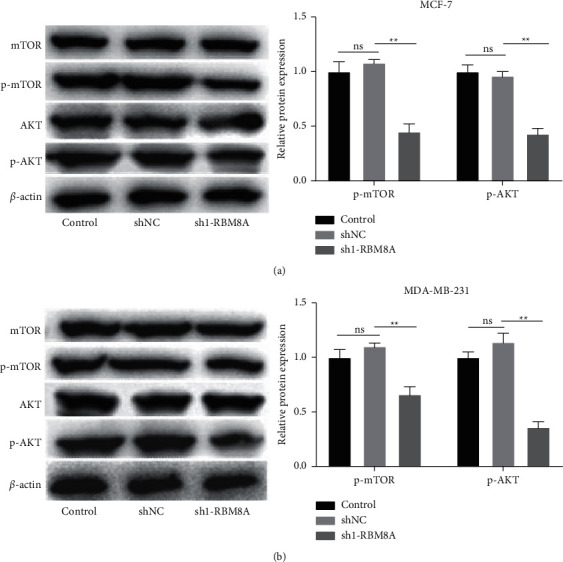
RBM8A silencing inhibits the AKT/mTOR pathway. (a and b) Western blot was performed to measure the protein expression of mTOR, phosphorylation (p)-mTOR, AKT, and p-AKT in MCF-7 and MDA-MB-231 cells. ^*∗∗*^*P* < 0.01 vs. sh-NC. Each experiment was repeated three times.

Afterward, the interaction between RBM8A silencing and AKT/mTOR pathway-related proteins was investigated. Following RBM8A silencing, the protein levels of p-AKT and p-mTOR were significantly declined in MCF-7 and MDA-MB-231 cells (*P* < 0.01, Figures 5(a)–5(b)), indicating that RBM8A silencing may inactivate the AKT/mTOR pathway in BC cells.

### 3.6. RBM8A Silencing Represses the Viability, DDP Resistance, and Metastasis of BC Cells via Inhibition of the AKT/mTOR Pathway

**Figure 6:**
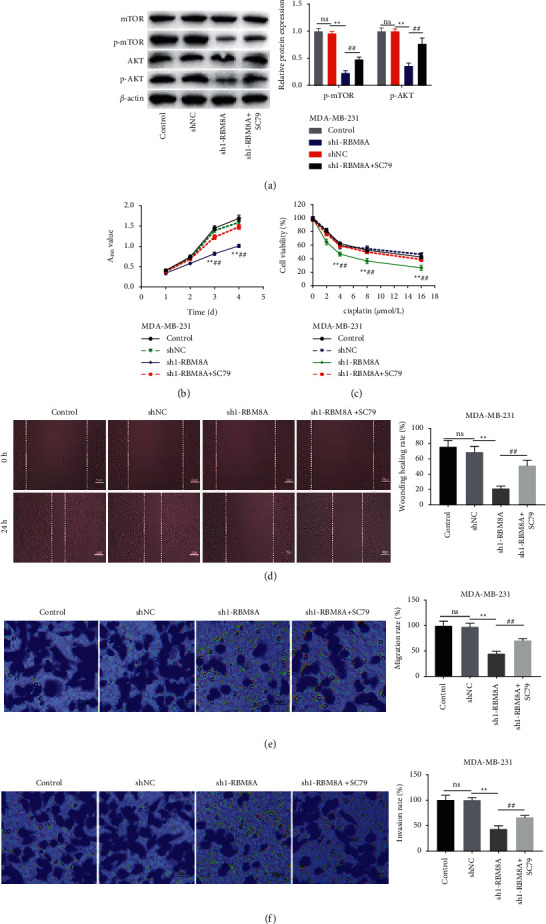
RBM8A silencing represses the viability, DDP resistance, and metastasis of breast cancer (BC) cells via inhibition of the AKT/mTOR pathway. (a) The protein expression of phosphorylation (p)-mTOR and p-AKT in MDA-MB-231 cells was detected by western blot. ^*∗∗*^*P* < 0.01 vs. sh-NC, ^##^*P* < 0.01 vs. sh1-RBM8A; (b and c) the viability and DDP resistance of MDA-MB-231 cells were detected by MTT assay. ^*∗∗*^*P* < 0.01 vs. sh-NC, ^##^*P* < 0.01 vs. sh1-RBM8A; (d) the migration of MDA-MB-231 cells was detected by wound-healing assay. (e and f) the migration and invasion of MDA-MB-231 cells were detected by transwell assay. ^*∗∗*^*P* < 0.01 vs. sh-NC, ^##^*P* < 0.01 vs. sh1-RBM8A. Each experiment was repeated three times.

We added the AKT activator SC79 to MDA-MB-231 cells to further verify whether RBM8A regulated the AKT/mTOR pathway in BC. As expected, the addition of SC79 reversed the inhibitory effects of RBM8A silencing on AKT/mTOR pathway-related proteins (*P* < 0.01, Figure 6(a)). SC79 partially reversed the inhibitory effects of RBM8A silencing on the viability and DDP resistance of MDA-MB-231 cells (*P* < 0.01, Figures 6(b)–6(c)), suggesting AKT signaling pathway mediates DDP resistance in MDA-MB-231 cells. Furthermore, the inhibitory effect of RBM8A silencing on the migration and invasion abilities of MDA-MB-231 cells was reversed by SC79 (*P* < 0.01, Figures 6(d)–6(f).

### 3.7. RBM8A Depletion Retards Tumor Growth of BC in the Mouse Model

**Figure 7:**
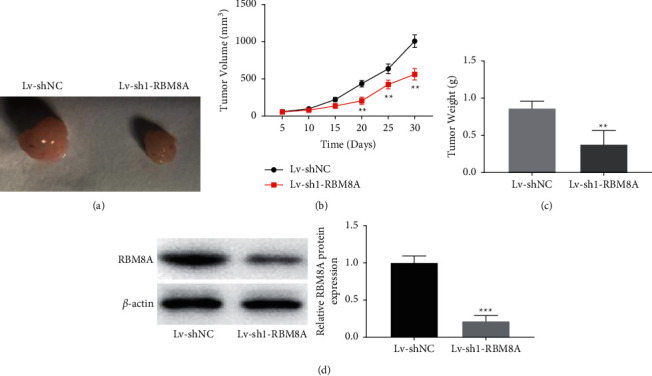
RBM8A depletion retards tumor growth of breast cancer (BC) in the mouse model. (a–c) The  effects of RBM8A knockdown on BC tumor volume and tumor weight were assessed. ^*∗∗*^*P* < 0.01 vs. Lv-sh-NC; (d) the protein expression of RBM8A was detected by western blot in xenograft tissues of BC. ^*∗∗∗*^*P* < 0.001 vs. Lv-sh-NC.

To test the function of RBM8A on BC *in vivo*, conducted tumor xenograft model was conducted. As shown in Figures 7(a)–7(c), the tumor weight and volume were significantly reduced in the Lv-sh1-RBM8A group compared with the Lv-sh-NC group (*P* < 0.01). Besides, the protein expression of RBM8A was dramatically reduced in tumor xenograft tissues of mice after injection of Lv-sh1-RBM8A (*P* < 0.001, Figure 7(d)).

## 4. Discussion

BC is a complex and heterogeneous disease [18]. Aberrations in gene expression are linked to BC pathogenesis. At present, the main treatment strategy for BC is surgery, supplemented by chemotherapy [19]. The increasing incidence and drug resistance of BC have put enormous pressure on clinical treatment [20]. Therefore, it is necessary to find new BC markers to improve the diagnosis, treatment, and prognosis of BC. In this work, we showed that overexpressed RBM8A promoted tumor cell growth in BC. In addition, RBM8A knockout in BC enhanced DDP sensitivity of BC cells.

Recently, increasing studies have pointed out that RBM8A exerts multiple biological functions. Aberrant expression of RBM8A has been detected in various malignancies, including hepatocellular carcinoma (HCC) and glioblastoma (GBM) [21, 22]. Abnormal expression of RBM8A is associated with carcinogenesis. RBM8A promotes tumor cell migration and invasion in HCC by activating the EMT signaling pathway *in vitro* [21]. Furthermore, it has been reported that RBM8A is up-regulated in GBM tissues, and its high expression is correlated with poor prognosis, while knockdown of RBM8A inhibits GBM progression and invasion [22]. Similar to the previous research, RBM8A expression was significantly increased in BC in the present study. RBM8A deletion suppressed BC cell viability, invasion, and migration, and promoted apoptosis. In addition, the high and low expression of RBM8A showed significant differences in TNM stage and LNM of BC. Similarly, LV et al. have reported that RBM8A expression is increased in gastric carcinoma tissues, and the level of RBM8A is correlated with tumor size, LNM, TNM stage, and distant metastasis in gastric cancer [11]. Therefore, we speculated that RBM8A may be an oncogene existing in BC to promote the progression of BC, which could be a promising biomarker and therapeutic target in the diagnosis and treatment of BC.

It is well known that DDP resistance is a critical problem in BC treatment, and the resistance to DDP in BC is generally evaluated by comparison of IC50. The repressed IC50 means the attenuated effect on the resistance to DDP of tumor cells [23]. Studies have reported that RBM8A is involved in the regulation of oxaliplatin resistance in HCC [9]. Based on previous research, we speculated that RBM8A may be an effective target to repress the development of BC at the cellular level. In the current study, RBM8A down-regulation not only decreased the cell viability as the concentrations of DPP are increased but also repressed the IC50 of DDP in MCF-7/DDP and MDA-MB-231/DDP cells. Therefore, we suggested that RBM8A knockdown may provide a therapeutic approach to enhance the sensitivity of BCs to DDP. To further explore the function of RBM8A in BC, the tumor xenograft model was established. As expected, animal experiments showed that RBM8A inhibition retarded the growth of tumor xenograft in mice. Collectively, we believed that the silencing of RBM8A may inhibit BC tumorigenesis via repression of the growth and metastasis of BC cells.

Studies have reported that the Akt/mTOR pathway plays a crucial role in essential cellular activities, such as cell proliferation, growth, and metabolism and found that it is commonly activated in human cancers [24]. The AKT/mTOR pathway is closely associated with BC tumorigenesis [25]. In this study, RBM8A knockdown suppressed the AKT/mTOR pathway activity in BC cells. The results indicated that RBM8A may interact with the AKT/mTOR pathway. To further confirm this conclusion, SC79 (an activator of AKT) was added to MDA-MB-231 to perform feedback experiments. We demonstrated that SC79 reversed the inhibiting effects of RBM8A knockdown on BC cell viability, invasion, and migration. Therefore, we suggested that RBM8A knockdown may retard the progression of BC via repression of the AKT/mTOR pathway. In addition, a growing number of studies have reported that the AKT/mTOR pathway is also a pivotal pathway involved in drug resistance regulation of BC [26]. For instance, Li et al. have indicated that LncRNA HOTAIR silencing attenuates the resistance of BC cells to DOX by inhibiting the AKT/mTOR pathway [27], suggesting that the AKT/mTOR pathway may be a key pathway to reverse DOX-resistance in BC. Studies have demonstrated that activation of the AKT/mTOR pathway enhances BC resistance to Adriamycin and promotes cancer development [28]. Moreover, Zong et al. have shown that the AKT/mTOR pathway is involved in cisplatin resistance induced by aerobic glycolysis in BC cells [29]. Consistent with previous studies, in the present study, SC79 reversed the inhibitory effect of RBM8A knockdown on DDP resistance in BC, pointing out that RBM8A knockdown may alleviate the resistance of BC cells to DDP by constraining AKT/mTOR pathway. In conclusion, RBM8A expression was up-regulated in BC tissues and cells. RBM8A may regulate proliferation, apoptosis, migration, and invasion, as well as promote DDP resistance, thereby affecting BC progression. Furthermore, RBM8A silencing decreased the DDP resistance in breast cancer cells by inhibiting the AKT/mTOR pathway. Our findings indicated that RBM8A has the potential to become a new therapeutic target for the treatment of BC.

## Figures and Tables

**Table 1:** Primers sequences.

Name of primer	Sequences (5′-3′)
RBM8A-F	GCGTGAGGATTATGACAGCGTG
RBM8A-R	TTCGGTGGCTTCCTCATGGACT
*β*-Actin-F	CTAAGGCCAACCGTGAAAAG
*β*-Actin-R	AACACAGCCTGGATGGCTAC

**Table 2:** Correlation between RBM8A expression and clinicopathological features in breast cancer patients.

Characteristics	Total *n* = 70	RBM8A expression		*P*-value
		Low (*n* = 35)	High (*n* = 35)	
Age				0.473
≤45	37	20	17	
>45	33	15	18	
Tumor size				0.334
≤2 cm	40	22	18	
>2 cm	30	13	17	
PR status				0.632
Negative	36	17	19	
Positive	34	18	16	
HER-2 status				0.626
Negative	42	20	22	
Positive	28	15	13	
TNM stage				0.004^*∗∗*^
I/II	38	13	25	
III/IV	32	22	10	
Lymph node metastasis				0.039^*∗*^
Negative	48	28	20	
Positive	22	7	15	

*Note.* ^*∗*^ *P* < 0.05, ^*∗∗*^*P* < 0.01. PR, progesterone receptor; HER-2, human epidermal growth factor receptor 2; TNM, tumor-node-metastases; RBM8A, RNA Binding Motif Protein 8A.

## Data Availability

All data generated or analyzed during this study are included within the published article.
